# Supplementary material for: Topological superconducting vortex from trivial electronic bands
Source: Nat Commun. 2023 Feb 6;14:640. doi: 10.1038/s41467-023-36347-w (PMC9902606; doi:10.1038/s41467-023-36347-w)
Supplement: Supplementary file 1 — Supplementary Information [file 41467_2023_36347_MOESM1_ESM.pdf]

Supplementary Information for "Topological Superconducting Vortex From Trivial Electronic Bands"

Lun-Hui Hu[1,2] and Rui-Xing Zhang[1,3,2,*]

[1]Department of Physics and Astronomy, The University of Tennessee, Knoxville, Tennessee 37996, USA
[2]Institute for Advanced Materials and Manufacturing, The University of Tennessee, Knoxville, Tennessee 37920, USA
[3]Department of Materials Science and Engineering, The University of Tennessee, Knoxville, Tennessee 37996, USA

(Dated: December 14, 2022)

CONTENTS

Supplementary Note 1: Topological Invariants of Vortex Lines 1
1.1 $\mathbb{Z}_2$ Topological Invariant 1
1.2 C_n Topological Charge 2

Supplementary Note 2: Vortex in Luttinger Semimetals: Numerical Results 3
2.1 Luttinger Semimetal from 6-band Kane Model 3
2.2 Bogoliubov-de Gennes Hamiltonian 4
2.3 Surface Local Density of States 6

Supplementary Note 3: Vortex Topology in Luttinger Semimetal: Analytical Theory 8

Supplementary Note 4: Continuum Model v.s. Lattice Model 11

References 11

Supplementary Note 1: Topological Invariants of Vortex Lines

In this part, we provide mathematical expressions of the $\mathbb{Z}_2$ and $\mathbb{Z}$-type topological invariants defined for the quasi one-dimensional (1D) vortex line Hamiltonian in the main text.

1.1 $\mathbb{Z}_2$ Topological Invariant

For C_n-symmetric vortex lines, $\mathbb{Z}_2$ topological invariant ν_{J_z} characterizes the gapped vortex-line topology of Caroli-de Gennes-Matricon (CdGM) states that belong to a particle-hole symmetry (PHS) invariant angular momentum sector, i.e., $J_z = 0$ or $J_z = n/2$ for spin-singlet s-wave pairing. Therefore, ν_{J_z} is exactly the $\mathbb{Z}_2$ topological invariant for 1D class D systems but with an additional J_z index. In this case, the quasi-1D system is always fully gapped without a topological phase transition. Following Ref. [1], under the Majorana basis, the vortex-line Hamiltonian matrix ${\mathcal{H}_{\mathcal{M}}}^{(J_z)}(k_z)$ for CdGM states in the J_z sector is antisymmetric and that is why its Pfaffian is well-defined. The $\mathbb{Z}_2$ topological invariant is thus defined as

$$\nu_{J_z} = \mathrm{sgn}\{\mathrm{Pf}[{\mathcal{H}_{\mathcal{M}}}^{(J_z)}(k_z = 0)]\}\mathrm{sgn}\{\mathrm{Pf}[{\mathcal{H}_{\mathcal{M}}}^{(J_z)}(k_z = \pi)]\} \in \mathbb{Z}_2. \tag{1}$$

The equivalence between the Pfaffian invariant in the Majorana representation and the quantized Berry phase of the occupied BdG bands in the Nambu basis has been established [2]. As a result, ν_{J_z} can be further expressed as

$$\nu_{J_z} = \frac{1}{2\pi}\mathrm{tr}\int_{-\pi}^{\pi} \mathcal{A}^{(J_z)}(k_z)dk_z, \tag{2}$$

where the non-Abelian Berry connection $\mathcal{A}_{nm}^{(J_z)}(k_z) = i\langle u_n^{(J_z)}|\partial_{k_z}|u_m^{(J_z)}\rangle$ is defined for all occupied CdGM bands carrying J_z. The above Berry phase formula can be further simplified if the 1D CdGM system features additional out-of-plane

mirror symmetry $\mathcal{M}_z$. Notice that the superconducting vortex line is aligned along z-axis. To unambiguously extract the value of ν_{J_z}, we just need the knowledge of the pattern of symmetry eigenvalues for the BdG occupied bands at high-symmetry momenta $k_z = 0, \pi$ [3]. In particular, let us define $m_{J_z,-}(0)$ and $m_{J_z,-}(\pi)$ as the number of occupied BdG bands at $k_z = 0$ and $k_z = \pi$, respectively, with a $\mathcal{M}_z = -1$ label, then we have

$$\nu_{J_z} = m_{J_z,-}(0) - m_{J_z,-}(\pi), \quad \mod 2. \tag{3}$$

This symmetry-based expression of ν_{J_z} aligns with the spirit of symmetry indicator theory.

1.2 C_n Topological Charge

In addition to the above $\mathbb{Z}_2$ topological invariant, we also define the C_n topological charge $\mathcal{Q}_{J_z} \in \mathbb{Z}$ that will indicate the number of symmetry-protected Dirac nodal crossings in the quasi-1D vortex-line spectrum. Our definition is similar to the topological charges defined for 3D Dirac semimetals [4] and for 3D Dirac superconductors [5].

Here, $\mathcal{Q}_{J_z}$ are defined for J_z sectors that are not PHS-invariant. PHS generally flips J_z to $-J_z$, forming a pair of PHS-related J_z sectors. Namely, if there exists a J_z-labeled CdGM state at k_z with an energy E, PHS mandates the existence of another partner state at $-k_z$ and energy $-E$, which is $-J_z$ labeled. The effective vortex Hamiltonian $h_{\text{vort}}(k_z)$ is generally gapped at $k_z = 0, \pi$ and we will focus on the occupied CdGM states with $E < 0$ at these high-symmetry momenta. Now we define $n_{J_z}^{(\alpha)}(k_i)$ as the number of occupied (unoccupied) J_z-labeled CdGM states at high-symmetry momentum $k_z = k_i$ (e.g., $k_i = 0, \pi$) with $\alpha = v$ ($\alpha = c$). The C_n symmetry charge is defined as

$$\mathcal{Q}_{J_z} \equiv n_{J_z}^{(v)}(0) - n_{J_z}^{(v)}(\pi), \tag{4}$$

for $J_z = 1$ for $C_{3,4}$ and $J_z = 1, 2$ for C_6. Because of the connectivity of energy bands,

$$n_{J_z}^{(c)}(0) + n_{J_z}^{(v)}(0) = n_{J_z}^{(c)}(\pi) + n_{J_z}^{(v)}(\pi). \tag{5}$$

Meanwhile, PHS requires that $n_{J_z}^{(c)}(k_i) = n_{-J_z}^{(v)}(k_i)$ and $n_{J_z}^{(v)}(k_i) = n_{-J_z}^{(c)}(k_i)$. It is then easy to show that equivalently,

$$\mathcal{Q}_{J_z} \equiv n_{J_z}^{(c)}(\pi) - n_{J_z}^{(c)}(0) = n_{-J_z}^{(c)}(0) - n_{-J_z}^{(c)}(\pi) = n_{-J_z}^{(v)}(\pi) - n_{-J_z}^{(v)}(0). \tag{6}$$

$|\mathcal{Q}_{J_z}|$ determines the number of C_n-protected 1D Dirac nodes from $k_z = 0$ to $k_z = \pi$, which is also the number of pairs of 1D Dirac nodes in the CdGM spectrum. These nodes can not be removed without (i) breaking C_n symmetry; and/or (ii) closing the energy gap at $k_z = 0, \pi$. As schematically shown in Fig. 1, the physical meaning of Eq. (4) can be understood as follows:

(i) Consider a PHS-related sector $(l, -l)$ and assume $n_l^{(v)}(0) = n, n_l^{(v)}(\pi) = m$, and $n_{-l}^{(v)}(0) = n'$. PHS requires $n_{-l}^{(c)}(0) = n, n_l^{(c)}(0) = n', n_{-l}^{(c)}(\pi) = m$.

(ii) Eq. (5) requires $n_l^{(c)}(\pi) = n_{-l}^{(v)}(\pi) = n + n' - m$.

(iii) To ensure the connectivity of the bands, there must be N_l number of l-indexed bands starting from the occupied bands at $k_z = 0$, crossing the zero energy, and ending at the conduction bands at $k_z = \pi$, where

$$N_l \equiv \mathcal{Q}_l = n_l^{(v)}(0) - n_l^{(v)}(\pi) = n - m. \tag{7}$$

Note that N_l is exactly our choice of C_n topological charge. Similarly, one can find $N_{-l} = n_{-l}^{(v)}(0) - n_{-l}^{(v)}(\pi) = m - n = -N_l$. If either N_l or N_{-l} is negative, this indicates the existence of left-moving modes along $\Gamma - Z$, instead of right-moving ones.

(iv) As a result, there are $|\mathcal{Q}_l|$ pairs of left-movers and right-movers along $\Gamma - Z$. They together form $|\mathcal{Q}_l|$ 1D Dirac nodes that are C_n-protected.

For the example shown in Fig. 1, we have $n = 4$ and $m = 2$, and this is why there are $|n - m| = 2$ Dirac nodes.

We note that when a pair of CdGM bands get inverted around a generic momentum $k_z \neq 0, \pi$, they can contribute to an additional pair of Dirac nodes that are not captured by $\mathcal{Q}_{J_z}$. These Dirac nodes, however, can be eliminated without closing the energy gap at $k_z = 0, \pi$. As a result, gapping these Dirac nodes will not lead to any topologically gapped state like a Kitaev vortex state. Therefore, we do not term vortices carrying these Dirac nodes as "topological" nodal vortex and we leave a discussion of these states to future works.

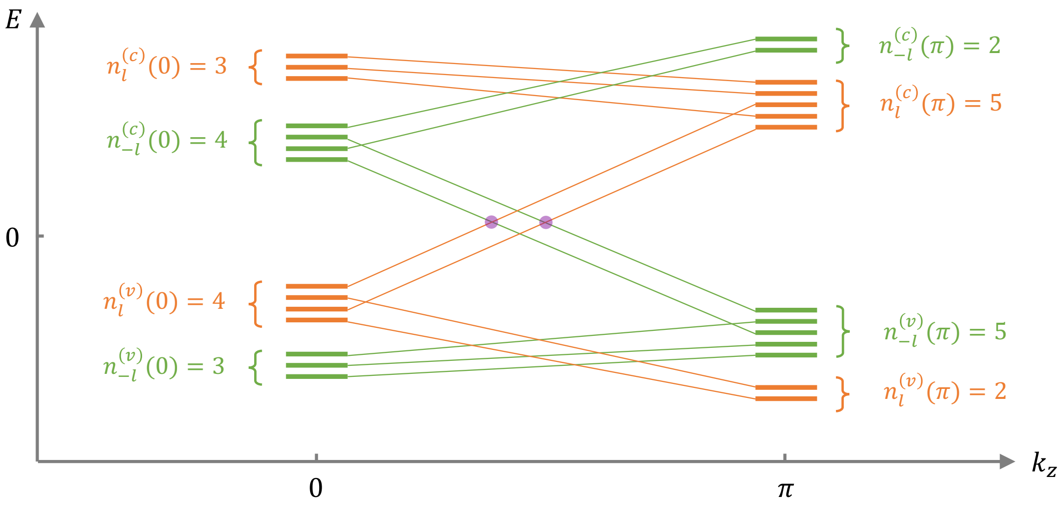

FIG. 1. Symmetry data counting and C_n topological charge $\mathcal{Q}_{J_z}$. The pattern of symmetry eigenvalues must satisfy both the particle-hole symmetry and the band connectivity relation. In the band configuration shown here, $\mathcal{Q}_l = n_l^{(v)}(0) - n_l^{(v)}(\pi) = 4 - 2 = 2$, indicating the existence of two 1D Dirac nodes denoted by the purple dots.

Supplementary Note 2: Vortex in Luttinger Semimetals: Numerical Results

In this section, we introduce the model Hamiltonian of a generalized Luttinger semimetal and study its vortex topological phase diagram.

2.1 Luttinger Semimetal from 6-band Kane Model

We first introduce the spin-$\frac{3}{2}$ matrices

$$J_x = \begin{pmatrix} 0 & \frac{\sqrt{3}}{2} & 0 & 0 \\ \frac{\sqrt{3}}{2} & 0 & 1 & 0 \\ 0 & 1 & 0 & \frac{\sqrt{3}}{2} \\ 0 & 0 & \frac{\sqrt{3}}{2} & 0 \end{pmatrix}, \quad J_y = \begin{pmatrix} 0 & -\frac{\sqrt{3}i}{2} & 0 & 0 \\ \frac{\sqrt{3}i}{2} & 0 & -i & 0 \\ 0 & i & 0 & -\frac{\sqrt{3}i}{2} \\ 0 & 0 & \frac{\sqrt{3}i}{2} & 0 \end{pmatrix}, \quad J_z = \begin{pmatrix} \frac{3}{2} & 0 & 0 & 0 \\ 0 & \frac{1}{2} & 0 & 0 \\ 0 & 0 & -\frac{1}{2} & 0 \\ 0 & 0 & 0 & -\frac{3}{2} \end{pmatrix}. \tag{8}$$

It is easy to check that $[J_i, J_j] = i\epsilon_{ijk}J_k$. Then the isotropic Luttinger Hamiltonian formed by the Γ_8 bands is

$$\begin{aligned} h_8(\mathbf{k}) &= (\lambda_1 + \frac{5}{2}\lambda_2)\mathbf{k}^2 - 2\lambda_2(\mathbf{k}\cdot\mathbf{J})^2 \\ &= -\lambda_1\mathbf{k}^2 + \begin{pmatrix} \lambda_2(k_x^2+k_y^2-2k_z^2) & -2\sqrt{3}\lambda_2 k_z k_- & -\sqrt{3}\lambda_2 k_-^2 & 0 \\ -2\sqrt{3}\lambda_2 k_z k_+ & -\lambda_2(k_x^2+k_y^2-2k_z^2) & 0 & -\sqrt{3}\lambda_2 k_-^2 \\ -\sqrt{3}\lambda_2 k_+^2 & 0 & -\lambda_2(k_x^2+k_y^2-2k_z^2) & 2\sqrt{3}\lambda_2 k_z k_- \\ 0 & -\sqrt{3}\lambda_2 k_+^2 & 2\sqrt{3}\lambda_2 k_z k_+ & \lambda_2(k_x^2+k_y^2-2k_z^2) \end{pmatrix}. \end{aligned} \tag{9}$$

The Hamiltonian form in terms of gamma matrices are defined in the main text. To fully incorporate the band topology of relevant quantum materials, we need to generalize the Luttinger model into a 6-band Kane model by including the Γ_6 bands. Therefore, we have

$$H_{\text{Kane}} = \begin{pmatrix} h_6(\mathbf{k}) & T(\mathbf{k}) \\ T^\dagger(\mathbf{k}) & h_8(\mathbf{k}) \end{pmatrix}, \tag{10}$$

where $h_6 = (E_c + \lambda_3 k^2)\sigma_0$ and

$$T(\mathbf{k}) = v\begin{pmatrix} -\frac{1}{\sqrt{2}}k_+ & \sqrt{\frac{2}{3}}k_z & \frac{1}{\sqrt{6}}k_- & 0 \\ 0 & -\frac{1}{\sqrt{6}}k_+ & \sqrt{\frac{2}{3}}k_z & \frac{1}{\sqrt{2}}k_- \end{pmatrix} \tag{11}$$

Note that H_{Kane} is essentially the same as $H_{\mathrm{Kane}}(1,\mathbf{k})$, but in a slightly different form. To identify the conditions for Luttinger semimetallic phase in the Kane model, we now project everything onto the Γ_8 bases, following

$$H_{\mathrm{eff}}(\mathbf{k}) = h_8 - T^\dagger h_6^{-1} T. \tag{12}$$

As required by the $O(3)$ symmetry, the effective Hamiltonian must take the same form as h_8 but the band parameters will get renormalized accordingly, where

$$\lambda_1 \to \lambda_1' = \lambda_1 + \frac{v^2}{3E_c}, \quad \lambda_2 \to \lambda_2' = \lambda_2 - \frac{v^2}{6E_c}. \tag{13}$$

In this case, the energy spectrum for H_{eff} is given by

$$\begin{aligned} E_{\frac{1}{2}} &= (-\lambda_1' + 2\lambda_2')k^2 = (-\lambda_1 + 2\lambda_2 - \frac{2v^2}{3E_c})k^2, \\ E_{\frac{3}{2}} &= (-\lambda_1' - 2\lambda_2')k^2 = (-\lambda_1 - 2\lambda_2)k^2. \end{aligned} \tag{14}$$

For HgTe-class materials, Γ_6 and Γ_8 bands are electron-like and hole-like, respectively, leading to $\lambda_1 > 0, \lambda_2 < 0, \lambda_3 > 0$ and $\lambda_1 > -2\lambda_2$. Meanwhile, the Γ_6-Γ_8 inversion requires $E_c < 0$. Therefore, to achieve a semimetallic phase, $E_{\frac{1}{2}}$ must play the role of electron bands and $E_{\frac{3}{2}}$ will be the hole bands. As a result, the LSM condition is

$$\begin{aligned} &\lambda_1 > 0,\ \lambda_2 < 0,\ \lambda_3 > 0,\ E_c < 0 \\ &-\lambda_1 - 2\lambda_2 < 0,\ -\lambda_1 + 2\lambda_2 - \frac{2v^2}{3E_c} > 0. \end{aligned} \tag{15}$$

Next, let us estimate the above projection parameters for the six-band Kane model, whose parameters are given by

$$\lambda_1 = \frac{4.1P^2}{18.8}, \lambda_2 = -\frac{0.5P^2}{18.8}, E_c = -0.303, \lambda_3 = \frac{P^2}{18.8}, v = P. \tag{16}$$

Note these parameters are all in unit of energy [eV]. Here $a_0 = 6.46$ Å is the in-plane lattice constant and $P = 8.46/a_0$. Now we use Eq. (14), the parameters for the projected LSM around the Γ point are given by

$$\lambda_1' = -1.51271, \lambda_2' = 0.897757. \tag{17}$$

Thus, the diagonal term for the $J_z = \pm 3/2$ bands reads

$$-(\lambda_1 + \frac{v^2}{3E_c})(k_x^2 + k_y^2 + k_z^2) + (\lambda_2 - \frac{v^2}{6E_c})(k_x^2 + k_y^2 - 2k_z^2) \approx 2.41(k_x^2 + k_y^2) - 3.3k_z^2. \tag{18}$$

We note that the sign is opposite with those for the LSM model used in the main text and Supplementary Note 2, where $-(k_x^2 + k_y^2) + 2k_z^2$ is used for the numerical simulation. This sign difference could directly give rise to the opposite vortex band dispersion between Fig. 1 and Fig. 3 in the main text. In summary,

$$\begin{cases} \text{Fig. 1: LSM model}, -(k_x^2 + k_y^2) + 2k_z^2, \text{the vortex-band with } J_z = +1 \text{ is a hole-like band}, \\ \text{Fig. 3: Kane model}, 2.41(k_x^2 + k_y^2) - 3.3k_z^2, \text{the vortex-band with } J_z = +1 \text{ is a electron-like band}. \end{cases} \tag{19}$$

The opposite effective mass due to the sign switching will be explicitly shown after deriving the low-energy vortex Hamiltonian from the perturbation theory in the Supplementary Note 3. However, the physics we want to address in the main text would not be affected (see Eq. 4). No matter m_1 is positive or negative, the vortex phase of a LSM is a Kitaev$\oplus$Nodal phase, thus, the vortex phase of a Kane model is nodal because of Eq. 4. Therefore, the choice of parameters of LSM completely does not affect our conclusion.

2.2 Bogoliubov-de Gennes Hamiltonian

We now discuss in details the Bogoliubov-de Gennes Hamiltonian of LSM and the numerical mapping of its vortex topological phase diagram. As shown in the main text, Hamiltonian for a general anisotropic LSM consists of four Γ_8 bands,

$$\mathcal{H}_{\mathrm{LSM}} = \lambda_1 k^2 \gamma_0 + M(\mathbf{k})\gamma_5 + v_z k_z (k_x \gamma_{45} + k_y \gamma_{35}) - \sqrt{3}\lambda_2((k_x^2 - k_y^2)\gamma_{25} + 2k_x k_y \gamma_{15}). \tag{20}$$

Here, $M(\mathbf{k}) = m_1(k_x^2 + k_y^2) + m_2 k_z^2$ and the 4×4 γ-matrices are defined as

$$\gamma_1 = \sigma_x \otimes s_z,\ \gamma_2 = \sigma_y \otimes s_z,\ \gamma_3 = \sigma_0 \otimes s_x,\ \gamma_4 = \sigma_0 \otimes s_y,\ \gamma_5 = \sigma_z \otimes s_z \tag{21}$$

with $\gamma_{mn} = -i\gamma_m\gamma_n$ and $\gamma_0 = \sigma_0 \otimes s_0$ the identity matrix. $\mathcal{H}_{\rm LSM}$ satisfies time-reversal symmetry $\Theta = i\gamma_{13}\mathcal{K}$ with $\mathcal{K}$ being the complex conjugate, as well as an out-of-plane mirror symmetry $\mathcal{M}_z = i\gamma_5$. Without loss of generality, we choose $\lambda_1 = 0$ for simplicity. Similar to the Kane model discussed in the main text, we further include the effect of lattice strain described by

$$\mathcal{H}_{\rm str} \triangleq \Sigma_{\rm str}\gamma_5 = \Sigma_{\rm str}\begin{pmatrix} 1 & & & \\ & -1 & & \\ & & -1 & \\ & & & 1 \end{pmatrix}. \tag{22}$$

$\Sigma_{\rm str}$ will become an important tuning parameter in our vortex topological phase diagram, as will be shown soon.

We now turn on an isotropic s-wave spin-singlet pairing potential and consider a vortex-line configuration along the z-axis. The corresponding Bogoliubov de-Gennes Hamiltonian (i.e., Eq. (1) in the main text) is given by

$$\mathcal{H}_{\rm BdG} = \begin{pmatrix} \mathcal{H}_{\rm LSM}(\mathbf{k}) - \mu & \mathcal{H}_\Delta \\ \mathcal{H}_\Delta^\dagger & \mu - \mathcal{H}^*_{\rm LSM}(-\mathbf{k}) \end{pmatrix}, \tag{23}$$

where μ is the chemical potential. The pairing function is captured by $\mathcal{H}_\Delta = i\Delta(\mathbf{r})\gamma_{13}$. Apparently, $\mathcal{H}_{\rm BdG}$ carries a trivial BdG bulk topology because of the s-wave pairing. The Nambu basis for $\mathcal{H}_{\rm BdG}$ is

$$|\Psi_{\rm BdG}\rangle = \left\{|\frac{3}{2}\uparrow\rangle_e, |\frac{1}{2}\downarrow\rangle_e, |-\frac{1}{2}\uparrow\rangle_e, |-\frac{3}{2}\downarrow\rangle_e, |\frac{3}{2}\uparrow\rangle_h, |\frac{1}{2}\downarrow\rangle_h, |-\frac{1}{2}\uparrow\rangle_h, |-\frac{3}{2}\downarrow\rangle_h\right\}^T, \tag{24}$$

where the atomic basis with a subscript e or h carries a crystal momentum $\mathbf{k}$ or $-\mathbf{k}$, respectively. In particular, the particle-hole symmetry

$$\Xi|J_z, s\rangle_e \to |-J_z, -s\rangle_h, \tag{25}$$

and a constant pairing term between $|J_z, s\rangle_e$ and $|J_z, s\rangle_h$ describes a spin-singlet s-wave Cooper pairing in our notation. Under this basis, we have

$$\mathcal{H}_{\rm BdG} = \begin{pmatrix} F_1+F_2 & vk_zk_- & \tilde{v}k_-^2 & 0 & \Delta e^{i\theta} & 0 & 0 & 0 \\ vk_zk_+ & F_1-F_2 & 0 & \tilde{v}k_-^2 & 0 & -\Delta e^{i\theta} & 0 & 0 \\ \tilde{v}k_+^2 & 0 & F_1-F_2 & -vk_zk_- & 0 & 0 & \Delta e^{i\theta} & 0 \\ 0 & \tilde{v}k_+^2 & -vk_zk_+ & F_1+F_2 & 0 & 0 & 0 & -\Delta e^{i\theta} \\ \Delta e^{-i\theta} & 0 & 0 & 0 & -F_1-F_2 & vk_zk_- & -\tilde{v}k_-^2 & 0 \\ 0 & -\Delta e^{-i\theta} & 0 & 0 & vk_zk_+ & -F_1+F_2 & 0 & -\tilde{v}k_-^2 \\ 0 & 0 & \Delta e^{-i\theta} & 0 & -\tilde{v}k_+^2 & 0 & -F_1+F_2 & -vk_zk_- \\ 0 & 0 & 0 & -\Delta e^{-i\theta} & 0 & -\tilde{v}k_+^2 & -vk_zk_+ & -F_1-F_2 \end{pmatrix}, \tag{26}$$

where $\tilde{v} = \sqrt{3}\lambda_2$ is used for short. Here (r, θ) describe the in-plane polar coordinates and k_z remains a good quantum number. The vortex line centering at $r = 0$ is described by $\Delta(\mathbf{r}) = \Delta_0 \tanh(r/\xi_0)e^{i\theta}$, where ξ_0 is the SC coherence length. Here $F_1 = \lambda_1\mathbf{k}^2 - \mu, F_2 = \Sigma_{\rm str} + \lambda_2(k_x^2 + k_y^2 - 2k_z^2)$. We define

$$\Xi = \tau_x\sigma_x s_x\mathcal{K} \triangleq \left(\begin{array}{cccc|cccc} 0&0&0&0&0&0&0&1 \\ 0&0&0&0&0&0&1&0 \\ 0&0&0&0&0&1&0&0 \\ 0&0&0&0&1&0&0&0 \\ \hline 0&0&0&1&0&0&0&0 \\ 0&0&1&0&0&0&0&0 \\ 0&1&0&0&0&0&0&0 \\ 1&0&0&0&0&0&0&0 \end{array}\right)\mathcal{K}, \quad U_{\rm BdG} = \begin{pmatrix} 1&0&0&0&0&0&0&0 \\ 0&0&0&0&0&0&1&0 \\ 0&0&0&0&1&0&0&0 \\ 0&0&1&0&0&0&0&0 \\ 0&1&0&0&0&0&0&0 \\ 0&0&0&0&0&0&0&1 \\ 0&0&0&0&0&1&0&0 \\ 0&0&0&1&0&0&0&0 \end{pmatrix}, \tag{27}$$

where Ξ is the operation of particle-hole symmetry (PHS). U_{BdG} is a unitary transformation transforming the original Nambu basis in Eq. (24) into a new basis $|\Psi_{\mathrm{BdG}}\rangle' = U_{\mathrm{BdG}}|\Psi_{\mathrm{BdG}}\rangle$, with

$$|\Psi_{\mathrm{BdG}}\rangle' = \left\{ |\frac{3}{2}\uparrow\rangle_e, |-\frac{1}{2}\uparrow\rangle_h, |\frac{3}{2}\uparrow\rangle_h, |-\frac{1}{2}\uparrow\rangle_e, |\frac{1}{2}\downarrow\rangle_e, |-\frac{3}{2}\downarrow\rangle_h, |\frac{1}{2}\downarrow\rangle_h, |-\frac{3}{2}\downarrow\rangle_e \right\}. \tag{28}$$

Under this transformation, the new Hamiltonian becomes

$$\mathcal{H}_{\mathrm{BdG}} = H_0(\mathbf{k}_\parallel) + H_1(\mathbf{k}_\parallel, k_z), \tag{29}$$

with

$$H_0(\mathbf{k}_\parallel) = \left(\begin{array}{cc|cc|cc|cc} 0 & 0 & \Delta e^{i\theta} & \tilde{v}k_-^2 & 0 & 0 & 0 & 0 \\ 0 & 0 & -\tilde{v}k_+^2 & \Delta e^{-i\theta} & 0 & 0 & 0 & 0 \\ \hline \Delta e^{-i\theta} & -\tilde{v}k_-^2 & 0 & 0 & 0 & 0 & 0 & 0 \\ \tilde{v}k_+^2 & \Delta e^{i\theta} & 0 & 0 & 0 & 0 & 0 & 0 \\ \hline 0 & 0 & 0 & 0 & 0 & 0 & -\Delta e^{i\theta} & \tilde{v}k_-^2 \\ 0 & 0 & 0 & 0 & 0 & 0 & -\tilde{v}k_+^2 & -\Delta e^{-i\theta} \\ \hline 0 & 0 & 0 & 0 & -\Delta e^{-i\theta} & -\tilde{v}k_-^2 & 0 & 0 \\ 0 & 0 & 0 & 0 & \tilde{v}k_+^2 & -\Delta e^{i\theta} & 0 & 0 \end{array}\right) \triangleq \begin{pmatrix} h_\Delta(\mathbf{k}_\parallel, \theta) & 0 \\ 0 & h_{-\Delta}(\mathbf{k}_\parallel, \theta) \end{pmatrix}. \tag{30}$$

and

$$H_1(\mathbf{k}_\parallel, k_z) = \left(\begin{array}{cc|cc|cc|cc} F_1+F_2 & 0 & 0 & 0 & vk_zk_- & 0 & 0 & 0 \\ 0 & -F_1+F_2 & 0 & 0 & 0 & -vk_zk_- & 0 & 0 \\ \hline 0 & 0 & -F_1-F_2 & 0 & 0 & 0 & vk_zk_- & 0 \\ 0 & 0 & 0 & F_1-F_2 & 0 & 0 & 0 & -vk_zk_- \\ \hline vk_zk_+ & 0 & 0 & 0 & F_1-F_2 & 0 & 0 & 0 \\ 0 & -vk_zk_+ & 0 & 0 & 0 & -F_1-F_2 & 0 & 0 \\ \hline 0 & 0 & vk_zk_+ & 0 & 0 & 0 & -F_1+F_2 & 0 \\ 0 & 0 & 0 & -vk_zk_+ & 0 & 0 & 0 & F_1+F_2 \end{array}\right). \tag{31}$$

2.3 Surface Local Density of States

The continuum LSM Hamiltonian can be regularized into a tight-binding (TB) model by replacing k_i, k_i^2 with $\sin(k_i), 2(1-\cos(k_i))$, respectively. Compared to the continuum Hamiltonian, the advantages of a TB model are

(1) it facilitates the studies of rotational symmetry breaking effects on the vortex-line topology.

(2) it allows for an iterative Green's function method to calculate the surface local density of states (LDOS) for a semi-infinite slab geometry.

In Fig. 2 of the main text, we have discussed two vortex topological phase diagrams for the isotropic Luttinger semimetal. Below, we briefly review the standard recursive Green's function method [6] to calculate the surface LDOS $D(\vec{r}_\parallel, \omega)$. The same methodology has been applied to generate similar surface LDOS plots for HgTe-class systems in Fig. 4 of the main text.

$$\begin{aligned} &\text{Step 1: initialize the in-plane vortex Hamiltonian } \mathcal{H}_\parallel \text{ and the z-direction hoping Hamiltonian } \mathcal{H}_z, \\ &\text{Step 2: initialize the first iteration, } T_{1,i} = \mathcal{H}_z, T_{2,i} = \mathcal{H}_z^\dagger, H_{1,i} = \mathcal{H}_\parallel, H_{2,i} = \mathcal{H}_\parallel, \\ &\text{Step 3: intermediate matrices, } A = [\omega + i\eta - H_{1,i}]^{-1}, M_1 = A \cdot T_{1,i}, M_2 = A \cdot T_{2,i}, \\ &\text{Step 4: the } (i+1)^{th} \text{ matrices, } T_{1,i+1} = T_{1,i} \cdot M_1, T_{2,i+1} = T_{2,i} \cdot M_2, \\ &\qquad\qquad H_{1,i+1} = H_{1,i} + T_{1,i} \cdot M_2 + T_{2,i} \cdot M_1, H_{2,i+1} = H_{2,i} + T_{1,i} \cdot M_2, \\ &\text{Step 5: after convergence (typically iteration number } N_{itr} \sim 13), G_n = [\omega + i\eta - H_{2,N_{itr}+1}]^{-1}, \\ &\text{Step 6: the surface Green's function } G_{\mathrm{surf}}(\vec{r}_\parallel, \omega) = [\omega + i\eta - \mathcal{H}_z \cdot G_n \cdot \mathcal{H}_z^\dagger]^{-1}. \end{aligned} \tag{32}$$

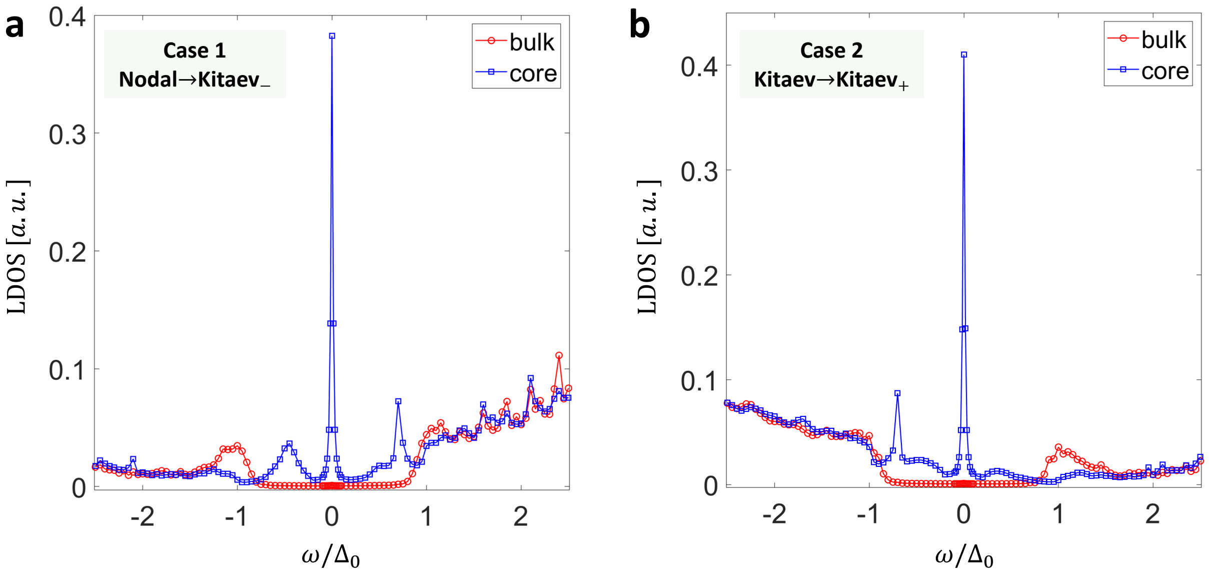

FIG. 2. Surface LDOS for Kitaev$_\pm$ vortex phases are shown in (a) and (b). The blue curve represents the LDOS $D_{\rm tot}(\vec{r}_c, \omega)$ at the vortex core center $\vec{r}_c = (20, 20)$ and the red curve is for the LDOS $D_{\rm tot}(\vec{r}_b, \omega)$ at a position $\vec{r}_b = (30, 30)$ far away from the vortex core. Here a.u. stands for arbitrary units.

Then the spin-resolved surface LDOS are defined as

$$D_{\rm tot}(\vec{r}_\parallel, \omega) = D_\uparrow(\vec{r}_\parallel, \omega) + D_\downarrow(\vec{r}_\parallel, \omega), \tag{33}$$

$$D_\uparrow(\vec{r}_\parallel, \omega) = -\frac{1}{\pi}{\rm Im}\left({\rm Tr}[M_\uparrow \cdot G_{\rm surf}(\vec{r}_\parallel, \omega)]\right), \tag{34}$$

$$D_\downarrow(\vec{r}_\parallel, \omega) = -\frac{1}{\pi}{\rm Im}\left({\rm Tr}[M_\downarrow \cdot G_{\rm surf}(\vec{r}_\parallel, \omega)]\right), \tag{35}$$

where $M_\uparrow$ and $M_\downarrow$ are the projection operator onto spin-up and spin-down subspace, respective. For the Luttinger semimetal model, they are

$$\begin{aligned} M_\uparrow &= \frac{\tau_0 + \tau_z}{2} \otimes \sigma_0 \otimes \frac{s_0 + s_z}{2}, \\ M_\downarrow &= \frac{\tau_0 + \tau_z}{2} \otimes \sigma_0 \otimes \frac{s_0 - s_z}{2}. \end{aligned} \tag{36}$$

Similarly, the spin projection operators for the six-band Kane model are

$$\begin{aligned} M_\uparrow &= \frac{\tau_0 + \tau_z}{2} \otimes {\rm Diag}[0, 1, 0, 1, 0, 1], \\ M_\downarrow &= \frac{\tau_0 + \tau_z}{2} \otimes {\rm Diag}[1, 0, 1, 0, 1, 0]. \end{aligned} \tag{37}$$

Vortex MZMs of both Kitaev$_-$ and Kitaev$_+$ vortex phases will induce pronounced zero-bias peaks (ZBPs) in both the total and spin-resolved LDOS at the vortex core. The simulation is performed on a 39×39 lattice with the following parameter set,

$$m_1 = -1, m_2 = 2, \lambda_1 = 0, \lambda_2 = -1, v_z = -2\sqrt{3}\lambda_2, \Delta_0 = 0.4, \Sigma_{\rm str} = 0.3, \Sigma_{\rm sb} = 0.2. \tag{38}$$

The Kitaev$_\pm$ vortex phases are achieved when $\mu = \mp 0.5$. We set the energy resolution $\eta = \Delta_0/80$ and show the numerical results in Fig. 2, where the blue curve represents the LDOS $D_{\rm tot}(\vec{r}_c, \omega)$ at the vortex core center $\vec{r}_c = (20, 20)$ and the red curve is for the LDOS $D_{\rm tot}(\vec{r}_b, \omega)$ at a position $\vec{r}_b = (30, 30)$ far away from the vortex core. As shown in Fig. 2, both Kitaev$_\pm$ phases show the significant zero-bias peak signature at vortex core center. This clearly demonstrates that vortex Majorana bound states can be indeed generated by doping a topologically trivial band insulator.

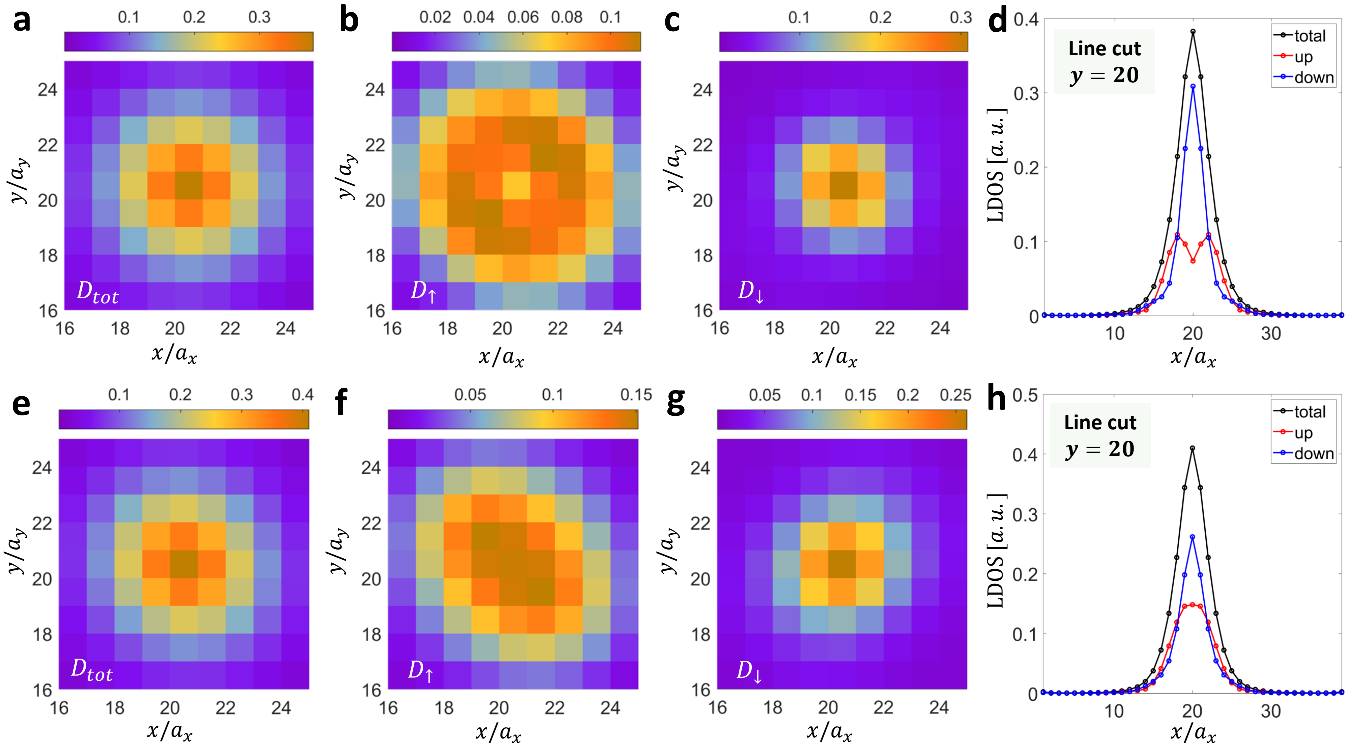

FIG. 3. The color map plots of the spin-resolved surface LDOS for Kitaev$_-$ and Kitaev$_+$ phases are shown in (a-d) and (e-h) near the vortex core center, respectively. The effect of rotation symmetry breaking is clearly visible. In particular, (d) and (h) shows the LDOS for Kitaev$_-$ and Kitaev$_+$ phases along the line cut with $y = 20$, both of which shows that the spin-down sector has a more pronounced zero-bias peak.

The spin-resolved LDOSs $D_\uparrow(\vec{r}_\parallel, \omega = 0)$ and $D_\downarrow(\vec{r}_\parallel, \omega = 0)$ at a zero bias voltage for both Kitaev$_-$ and Kitaev$_+$ vortex phases are shown in Fig. 3, where (a-d) is for the Kitaev$_-$ vortex and (e-h) is for the Kitaev$_+$ vortex. The C_2-symmetric vortex profile images in Fig. 3 (a) and (e) confirm the breaking of rotational symmetry by Σ_{sb}, which should be experimentally detectable. In addition, we also notice that both cases show that $D_\uparrow(\vec{r}_c, \omega = 0) < D_\downarrow(\vec{r}_c, \omega = 0)$ at the vortex core center $\vec{r}_c = (20, 20)$, which is consistent with the LDOSs of the Kitaev$_-$ vortex phase of the six-band Kane model [see Fig. 4 in the main text]. This is reasonable since the Kitaev$_-$ vortex in the Kane model originates from its LSM physics.

Supplementary Note 3: Vortex Topology in Luttinger Semimetal: Analytical Theory

In this part, we will combine both analytical and numerical expertise to show that how $H_1(\mathbf{k})$ in Eq. (31) will make the zero modes disperse k_z and further develop vortex-line topology in the superconducting Luttinger semimetal.

The chiral winding number calculation described in Methods is powerful to analytically prove the existence of four zero modes. For our purpose, we will need additional information of the zero-mode wavefunction, which turns out to be analytically challenging to obtain. Instead, we choose to extract the general form of the zero-mode wavefunctions through a large-scale numerical calculation. In particular, we find that two of the zero modes carry $J_z = 0$ and the

other two belong to the $J_z = \pm 1$ sector:

$$
\begin{aligned}
|\Phi(0,r,\theta)\rangle_1 &= \Big\{u_1(-1,r)e^{-i\theta}, u_2(0,r), 0,0,0,0,0,0\Big\}^T,\\
|\Phi(0,r,\theta)\rangle_2 &= \Big\{0,0,0,0,u_2(0,r), u_1(-1,r)e^{i\theta}, 0,0\Big\}^T,\\
|\Phi(1,r,\theta)\rangle_1 &= \Big\{u_1(0,r), u_2(1,r)e^{i\theta}, 0,0,0,0,0,0\Big\}^T,\\
|\Phi(-1,r,\theta)\rangle_2 &= \Big\{0,0,0,0,u_2(1,r)e^{-i\theta}, u_1(0,r), 0,0\Big\}^T.
\end{aligned}
\tag{39}
$$

Here $u_1(-1,r), u_2(0,r)$ and $u_1(0,r), u_2(1,r)$ follow the definition in Methods in the main text and their coefficients can be determined numerically. As will be shown next, these coefficient details do not matter in terms of the vortex topological conclusion.

The four zero modes in Eq. (39) span the following low-energy basis function that is crucial for understanding vortex topology,

$$
|\Psi_{\text{vortex}}\rangle = \{|\Phi(0,r,\theta)\rangle_1, |\Phi(0,r,\theta)\rangle_2, |\Phi(1,r,\theta)\rangle_1, |\Phi(-1,r,\theta)\rangle_2\}. \tag{40}
$$

Projecting $H_1(\mathbf{k})$ onto the zero-mode manifold, we arrive at a 4 by 4 vortex Hamiltonian consisting of two 2 by 2 decoupled blocks,

$$
h_{\text{vortex}} = \langle\Psi_{\text{vortex}}|H_1(\boldsymbol{k}_\parallel, k_z)|\Psi_{\text{vortex}}\rangle = \left(\begin{array}{c|c} h_{\text{Kitaev}} & 0 \\ \hline 0 & h_{\text{Nodal}} \end{array}\right). \tag{41}
$$

In particular, we find that

$$
h_{\text{Kitaev}} = \begin{pmatrix} K_0 + K_z k_z^2 & K_v k_z \\ K_v k_z & -K_0 - K_z k_z^2 \end{pmatrix}, \tag{42}
$$

$$
h_{\text{Nodal}} = \begin{pmatrix} N_0 + N_z k_z^2 & 0 \\ 0 & -N_0 - N_z k_z^2 \end{pmatrix}, \tag{43}
$$

with

$$
K_0 = K_\mu + K_\parallel + K_\Sigma, \quad N_0 = N_\mu + N_\parallel + N_\Sigma. \tag{44}
$$

The explicit form of each projection coefficient is given by

$$
K_v = v \times \int_0^{2\pi} \frac{d\theta}{2\pi} \int_0^{R_{\text{disk}}} rdr\ \left[(u_1^*(-1,r)e^{i\theta})[k_-](u_2(0,r)) + (u_2^*(0,r))[k_-](u_1(1,r)e^{i\theta})\right], \tag{45}
$$

$$
K_\mu = \mu \times \int_0^{2\pi} \frac{d\theta}{2\pi} \int_0^{R_{\text{disk}}} rdr\ \left[|u_1(-1,r)|^2 - |u_2(0,r)|^2\right], \tag{46}
$$

$$
K_\parallel = m_1 \times \int_0^{2\pi} \frac{d\theta}{2\pi} \int_0^{R_{\text{disk}}} rdr\ \left[(u_1^*(-1,r)e^{i\theta})[k_+k_-](u_1(-1,r)e^{-i\theta}) + (u_2^*(0,r))[k_+k_-](u_2(0,r))\right], \tag{47}
$$

$$
K_z = m_2 \times \int_0^{2\pi} \frac{d\theta}{2\pi} \int_0^{R_{\text{disk}}} rdr\ \left[|u_1(-1,r)|^2 + |u_2(0,r)|^2\right], \tag{48}
$$

$$
K_\Sigma = \Sigma_{\text{str}} \times \int_0^{2\pi} \frac{d\theta}{2\pi} \int_0^{R_{\text{disk}}} rdr\ \left[|u_1(-1,r)|^2 + |u_2(0,r)|^2\right], \tag{49}
$$

$$
N_\mu = \mu \times \int_0^{2\pi} \frac{d\theta}{2\pi} \int_0^{R_{\text{disk}}} rdr\ \left[|u_1(0,r)|^2 - |u_2(1,r)|^2\right], \tag{50}
$$

$$
N_\parallel = m_1 \times \int_0^{2\pi} \frac{d\theta}{2\pi} \int_0^{R_{\text{disk}}} rdr\ \left[(u_1^*(0,r))[k_+k_-](u_1(0,r)) + (u_2^*(1,r)e^{-i\theta})[k_+k_-](u_2(1,r)e^{i\theta})\right], \tag{51}
$$

$$
N_z = m_2 \times \int_0^{2\pi} \frac{d\theta}{2\pi} \int_0^{R_{\text{disk}}} rdr\ \left[|u_1(0,r)|^2 + |u_2(1,r)|^2\right], \tag{52}
$$

$$
N_\Sigma = \Sigma_{\text{str}} \times \int_0^{2\pi} \frac{d\theta}{2\pi} \int_0^{R_{\text{disk}}} rdr\ \left[|u_1(0,r)|^2 + |u_2(1,r)|^2\right]. \tag{53}
$$

where we set $F_1 = 0$ and $F_2 = \Sigma_{\rm str} + m_1(k_x^2 + k_y^2) + m_2 k_z^2$ for simplicity and the relation $u(-1, r) = -u(1, r)$ has been applied because of $J_n(r) = (-1)^n J_{-n}(r)$.

To prove that $h_{\rm Kitaev}$ and $h_{\rm Nodal}$ describe a Kitaev vortex and a nodal vortex, respectively, we first note that

$$\mathrm{sgn}[K_\Sigma] = \mathrm{sgn}[N_\Sigma] = \mathrm{sgn}[\Sigma_{\rm str}] \tag{54}$$

We now prove the following relations:

$$\mathrm{sgn}[K_\parallel] = \mathrm{sgn}[N_\parallel] = \mathrm{sgn}[m_1], \quad \mathrm{sgn}[K_z] = \mathrm{sgn}[N_z] = \mathrm{sgn}[m_2]. \tag{55}$$

Take $K_\parallel$ as an example and we need to evaluate the integral into two parts:

- $u_2(0, r)$: According to the Bessel function expansion in Methods, we have

$$u_2(0, r) = \sum_{j=1}^{N} c_{j,0}\phi(0, r, \alpha_j), \tag{56}$$

  which gives rise to

$$\begin{aligned}
&\int_0^{2\pi} \frac{d\theta}{2\pi} \int_0^{R_{\rm disk}} rdr\ [(u_2^*(0,r))[k_+k_-](u_2(0,r))] \\
&= \int_0^{2\pi} \frac{d\theta}{2\pi} \int_0^{R_{\rm disk}} rdr \left[(\sum_{j=1}^{N} c_{j,0}^*\phi(0,r,\alpha_j))[k_+k_-](\sum_{l=1}^{N} c_{l,0}\phi(0,r,\alpha_l)) \right] \\
&= \sum_{j=1}^{N}\sum_{l=1}^{N} c_{j,0}^* \times c_{l,0} \times \frac{\alpha_l^2}{R_{\rm disk}^2} \times \left[\int_0^{R_{\rm disk}} rdr\ \phi(0,r,\alpha_j)\phi(0,r,\alpha_l) \right] \\
&= \sum_{j=1}^{N}\sum_{l=1}^{N} c_{j,0}^* \times c_{l,0} \times \frac{\alpha_l^2}{R_{\rm disk}^2} \times \delta_{j,l} \\
&= \sum_{j=1}^{N} |c_{j,0}|^2 \times \frac{\alpha_j^2}{R_{\rm disk}^2} \geq 0.
\end{aligned} \tag{57}$$

  Here, α_j is the zero of $\mathcal{J}_0(r)$ and we have used the fact that $[k_+k_-]\phi(0, r, \alpha_l) = \frac{\alpha_l^2}{R_{\rm disk}^2}$.

- $u_1(-1, r)$: Similarly, we can easily prove that

$$\int_0^{2\pi} \frac{d\theta}{2\pi} \int_0^{R_{\rm disk}} rdr\ \left[(u_1^*(-1,r)e^{i\theta})[k_+k_-](u_1(-1,r)e^{-i\theta})\right] = \sum_{j=1}^{N} |c_{j,-1}|^2 \times \frac{\alpha_j^2}{R_{\rm disk}^2} \geq 0, \tag{58}$$

  where α_j is the zero of $\mathcal{J}_1(r)$.

Combining the above two contributions together, we have proved that

$$K_\parallel = m_1[\sum_{j=1}^{N}(|c_{j,0}|^2 + |c_{j,-1}|^2) \times \frac{\alpha_j^2}{R_{\rm disk}^2}], \tag{59}$$

and clearly $\mathrm{sgn}(K_\parallel) = \mathrm{sgn}(m_1)$. Similarly, we can prove the other relations in Eq. (55). This complete our proof of the nontrivial topological properties of vortex Hamiltonian in Eq. (41). For LSM, $m_1 = \lambda_2 < 0$ and $m_2 = -2\lambda_2 > 0$. With $\mu = \Sigma_{\rm str} = 0$, we have

$$K_\parallel K_z < 0, \quad N_\parallel N_z < 0. \tag{60}$$

This immediately indicates the inverted band structure for both $h_{\rm Kitaev}$ and $h_{\rm Nodal}$, leading to

$$\nu_0 = 1, \mathcal{Q}_1 = 1. \tag{61}$$

The above topological invariants explain the coexistence of Kitaev vortex and nodal vortex for LSM. The mapping between bulk and vortex coefficients further allow us to qualiatively understand the strain-induced vortex topological phase diagram. For example, a negative $\Sigma_{\rm str} < 0$ enhances the vortex-mode band inversion and further stabilizes the Kitaev$\oplus$nodal vortex phase. A positive $\Sigma_{\rm str}$, however, weakens the vortex topology and make other phases in the VTPD (e.g. Kitaev, nodal, trivial vortex phases) to emerge.

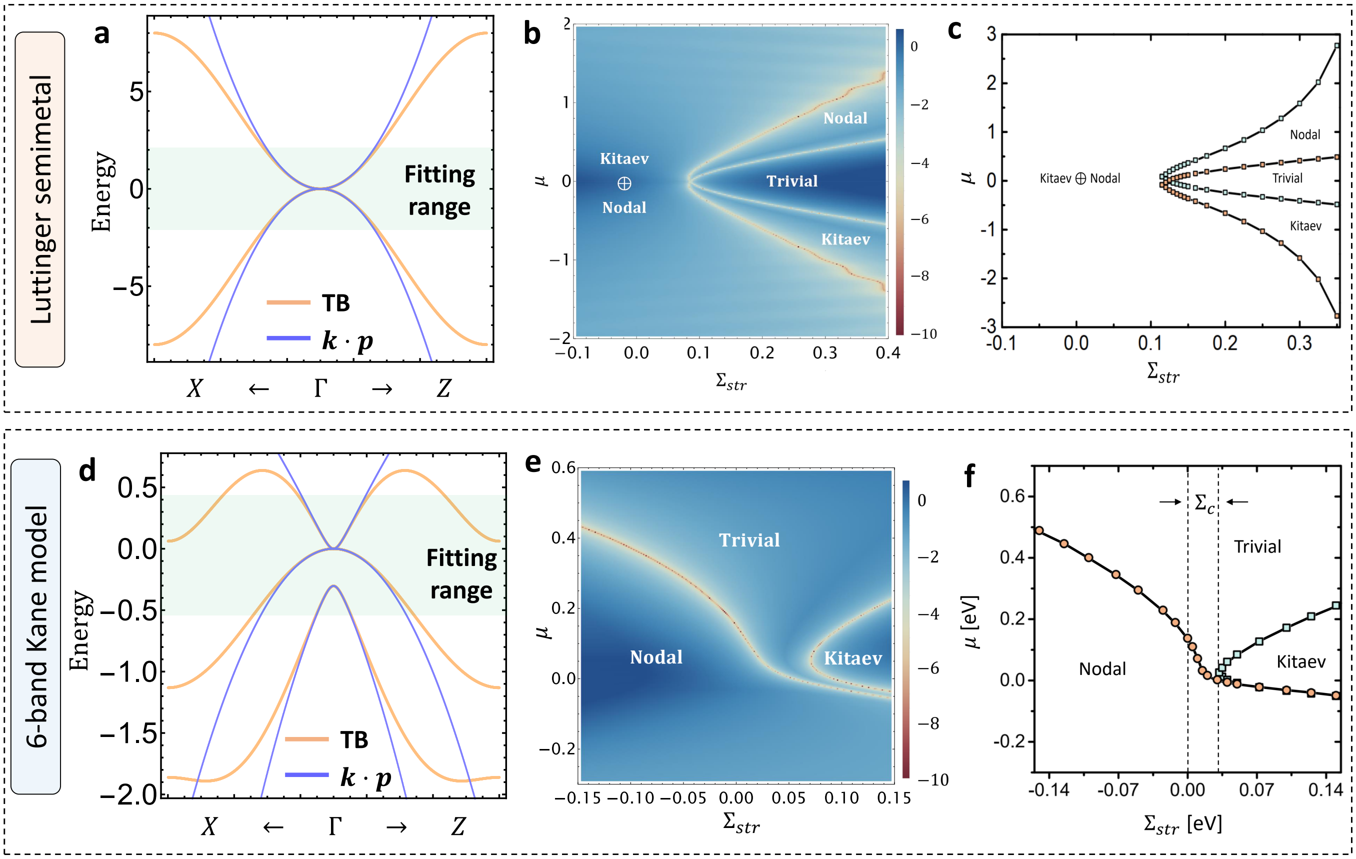

FIG. 4. Continuum models v.s. lattice models. (a) and (d) are the bulk band structures, where the green shaded region denotes the energy range where continuum and lattice models fit well with each other. Clearly, within these energy windows, the vortex topological phase diagrams (VTPDs) shown in (b) and (c), as well as in (e) and (f), also agree well. Here (b) and (e) are obtained from the tight-binding models, while (c) and (f) are based on the continuum models. For the results based on tight-binding model, the VTPDs are achieved by mapping out the vortex energy gap at $k_z = 0$, whose logarithmic value is shown by the colors in (b) and (e).

Supplementary Note 4: Continuum Model v.s. Lattice Model

In Fig. 4, we provide a comprehensive comparison between the lattice model and the continuum $\mathbf{k} \cdot \mathbf{p}$ model for both LSM and HgTe, including bulk band structures and vortex topological phase digrams. The lattice models are regularized on a in-plane square lattice, with k_z still being a good quantum number. Clearly, the results from both lattice and continuum models agrees well in a quantitative manner, for both LSM model and Kane model. Therefore, the main conclusions of our work are robust and do not depend on the explicit types of models that are adopted in the numerical simulations.

* ruixing@utk.edu

[1] A. Y. Kitaev, Physics-Uspekhi **44**, 131 (2001).
[2] J. C. Budich and E. Ardonne, Phys. Rev. B **88**, 075419 (2013).
[3] A. Alexandradinata, X. Dai, and B. A. Bernevig, Phys. Rev. B **89**, 155114 (2014).
[4] B.-J. Yang, T. Morimoto, and A. Furusaki, Phys. Rev. B **92**, 165120 (2015).
[5] R.-X. Zhang, Y.-T. Hsu, and S. Das Sarma, Phys. Rev. B **102**, 094503 (2020).
[6] M. L. Sancho, J. L. Sancho, J. L. Sancho, and J. Rubio, Journal of Physics F: Metal Physics **15**, 851 (1985).
